# Online Profile of Canadian Diagnostic Radiology Residents: Do Residents Alter Their Profile When Applying for Fellowships?

**DOI:** 10.15694/mep.2018.0000102.1

**Published:** 2018-05-16

**Authors:** Anthony Vo, Arlene Kanigan

**Affiliations:** 1 University of Alberta

**Keywords:** social media, residents, fellowship, Canadian, radiology

## Abstract

This article was migrated. The article was marked as recommended.

**Objective:** Survey the online profile of Canadian Diagnostic Radiology residents at the University of Alberta and determine whether residents alter it when applying for fellowships, due to the perceived assessment of their profile by Fellowship Selection Committees.

**Methods:** A cross-sectional study was performed by distributing an anonymous questionnaire to 31 residents at the University of Alberta. Descriptive and ANOVA statistical analyses were performed. P-value less than 0.01 was considered statistically significant.

**Results:** 26 questionnaires were completed. The average age was 28.9. 91.4% of residents have Facebook, followed by Instagram (30.4%) and ResearchGate (30.4%). 52.5% viewed their profile at least once daily, although 83.3% make changes to it less than once per month. The profiles were primarily for personal use (72.7%) and none were solely for professional use. 53.8% felt that Fellowship Selection Committees assess their profile and 69.2% were neutral or agreed with this. In anticipation, 70.6% would restrict profile viewership, while 29.4% would change their profile name, predominantly due to the sensitive and personal information. 92.8% would make the changes at least 2 months prior to the application deadline. There was no statistical difference between age and having a profile (p=0.597), agreement with Fellowship Selection Committees using a resident’s profile for selection (p=0.91), how often residents viewed (p=0.827) or changed (p=0.934) their profile.

**Conclusion:** Nearly all Canadian residents at our institution have an online profile and over half view it at least once daily. The majority of residents perceived that their profile is assessed by Fellowship Selection Committees, but are not against it. In anticipation, most residents would alter their profile prior to the application deadline mainly due to the sensitive and personal information.

## Introduction

Social media is becoming more prevalent within our modern society. The Pew Internet and American Life Project (2018) showed that the use of social media has increased from 8% in 2005 to 76% in 2015. Some of the well-known platforms used include Facebook (Menlo Park, California, U.S.A.), Instagram (Menlo Park, California, U.S.A.), and Twitter (San Francisco, California, U.S.A.). In medicine, social media is commonly used among trainees, with a prevalence of greater than 90% among medical students and 91% among Radiology trainees ( Capps, 2017).

Social media can be used for personal and/or for professional purposes. From communicating and sharing important life events with family members to disseminating and increasing visibility of academic research or endeavours with colleagues. By doing so, an individual creates an “online profile” of themselves, which can provide others with information about themselves. However, that information may not always conform to the individual’s professional conduct. In a study by Chretien (2009), they found that 60% of U.S.A. allopathic medical schools have reported incidents of students posting unprofessional content online. This may have prognostic significance given that a positive correlation between unprofessional behavior in medical school and future state medical board disciplinary action was found ( Papadakis, 2005). These have contributed to the use of an applicant’s online profile as a means of assessing their clinical competence and professional accountability by certain postgraduate medical residency programs ( Thompson, 2008). In surgical specialties, 17% of U.S.A. program directors screened applicants using their online profile, and 33% of this group gave lower rankings to applicants based on what they found (Go, 2017). With the increasing number of faculty members who live in an era of social media, the reception of online profile and its use as an evaluation tool, will likely increase ( Go, 2012). Consequently, as more medical trainees perceive the use of their online profile as part of the selection process, they may alter it to counteract this. In a study by Strausburg (2013) they found that the majority of medical students altered or planned to alter their online profile in preparation for the residency match.

The process of evaluating a candidate’s application traditionally relied on academic achievements, however nonacademic qualities are becoming increasingly important ( Thompson, 2008). This can be problematic for postgraduate medical residents applying to fellowship programs because they don’t often have the same opportunities they had as medical students, such as the lack of clinical electives at programs they are applying to. Therefore, the ability of the fellowship program to assess the nonacademic qualities of the applicants is difficult. This may influence Fellowship Selection Committees to put greater emphasis on what is available to them, including an applicant’s online profile. Given that the prevalence of online profile, its pattern of consumption and its use may vary based on differences in culture and attitude, it is important to review the Canadian literature, to determine whether this would be an effective use of resources by these committees. Unfortunately, there is a paucity of Canadian literature regarding the use of social media among residents, especially those in Diagnostic Radiology. Furthermore, the residents’ perception of whether their online profile is assessed by Fellowship Selection Committees when they apply and their behavior when applying has not been well described.

Therefore, the primary objective of the study was to survey the online profile of Canadian Diagnostic Radiology residents at the University of Alberta. The secondary objective was to determine whether residents alter their online profile when applying for fellowships, due to the perceived assessment of their profile by Fellowship Selection Committees.

## Methods

Following a review of the literature, an anonymous online questionnaire was devised using Google Forms (Menlo Park, California,U.S.A). The initial questionnaire was piloted by two residents and suggestions and feedback were then incorporated. The final questionnaire consisted of 15 questions consisting of dichotomous, rating scale, multiple choice, and open-ended questions. The questionnaire was then distributed to Diagnostic Radiology residents at the University of Alberta, Canada, in late 2017 using an e-mail list. The questionnaire was available for 2 weeks with one e-mail reminder in between. Participants voluntarily completed the questionnaire by clicking a provided link. No identifiable information was collected. After 2 weeks, the anonymous data was evaluated using descriptive and analysis of the variance (ANOVA) statistical analyses. Statistical significance was defined as a p-value less than 0.01. The Department’s Research Coordinator deemed this project to be a pilot/feasibility study that followed the principles of the Declaration of Helsinki and so did not require formal ethical approval.

## Results

**Figure 1.**
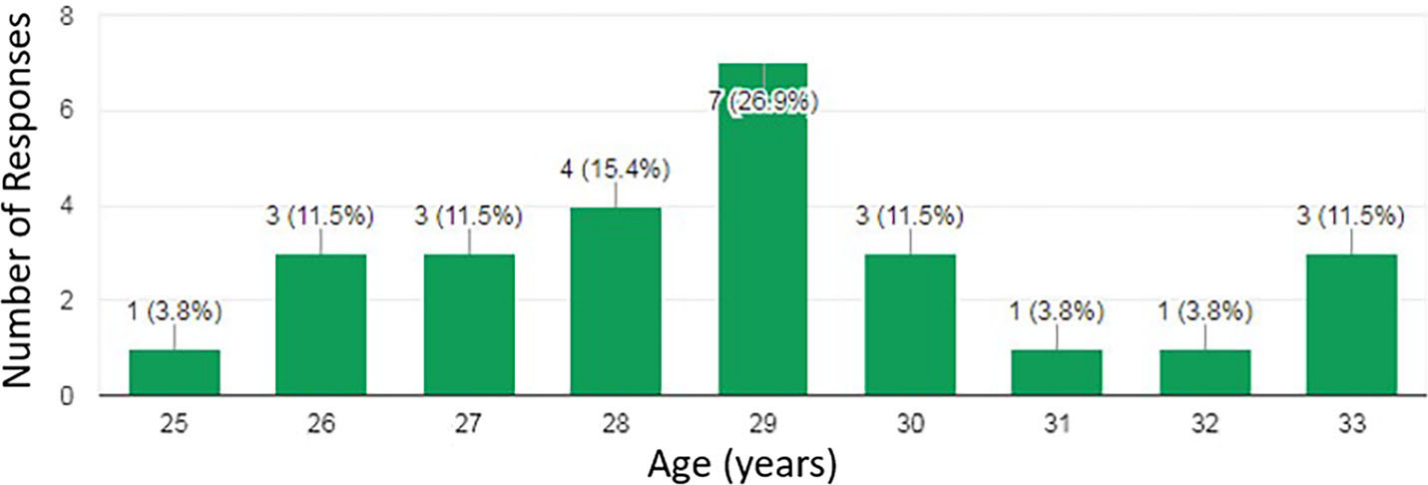
Age distribution of respondents.

**Figure 2.**
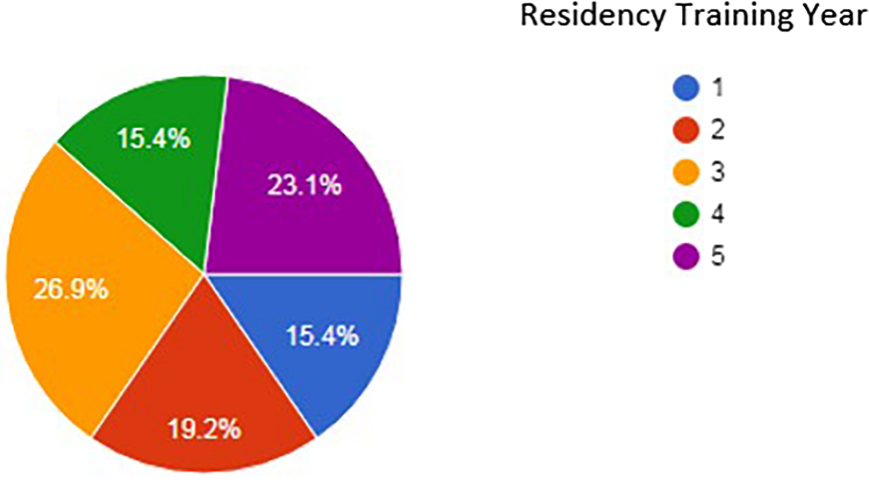
Percentage of respondents in each Diagnostic Radiology residency training year at the University of Alberta.

**Figure 3.**
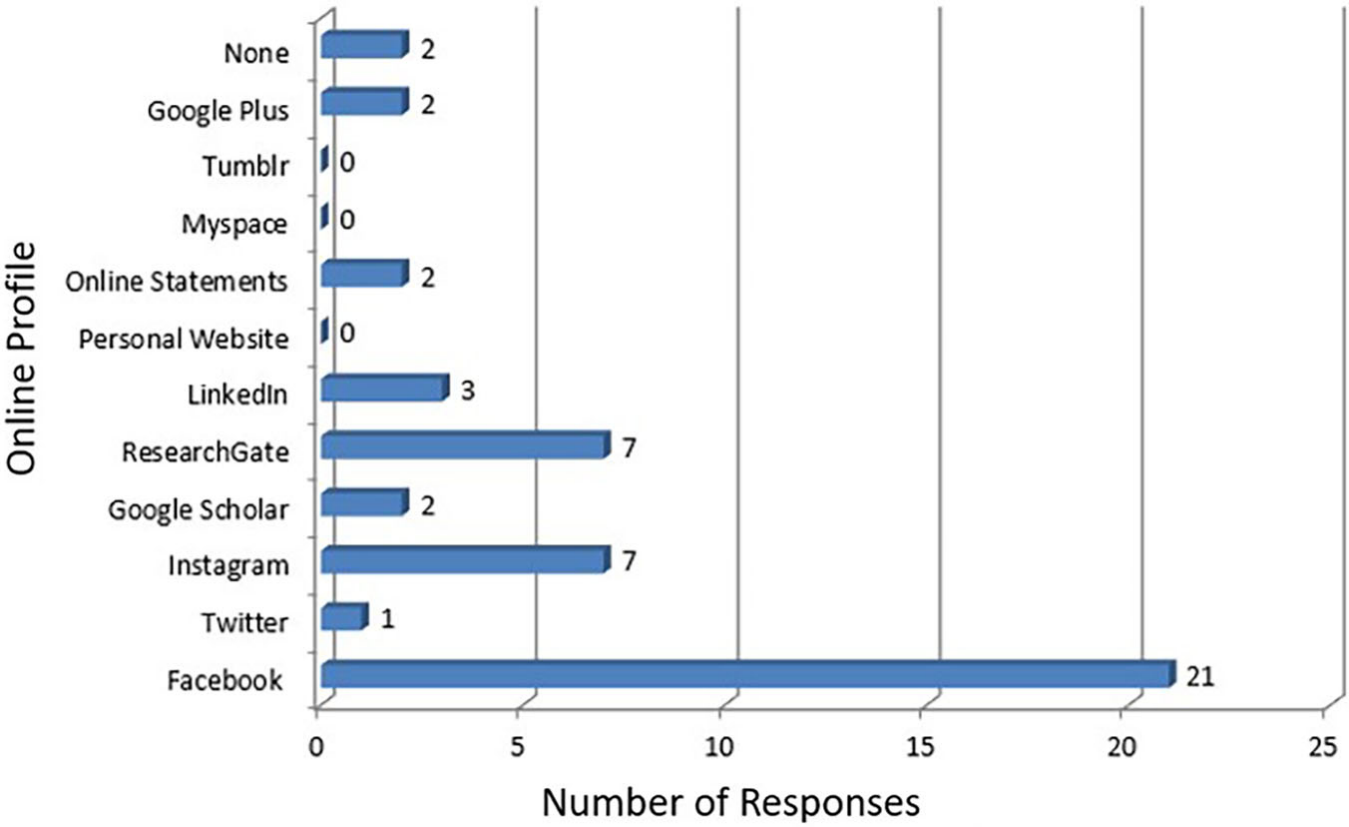
Online profile of the residents.

83.9% of questionnaires were completed (n=26). The average age was 28.9 years ( Figure 1). There was no statistical difference between age and having an online profile (p=0.597). The majority of residents were male (80.8%). There were respondents throughout all 5 years of the residency training program ( Figure 2). Almost all residents had a Facebook profile (91.3%, n=21). This was followed by a tie between Instagram (30.4%, n=7) and ResearchGate (30.4%, n=7) ( Figure 3). Their profile was primarily used for personal use only (72.7%). 27.3% were used for both personal and professional use. None were solely for professional use.

**Figure 4.**
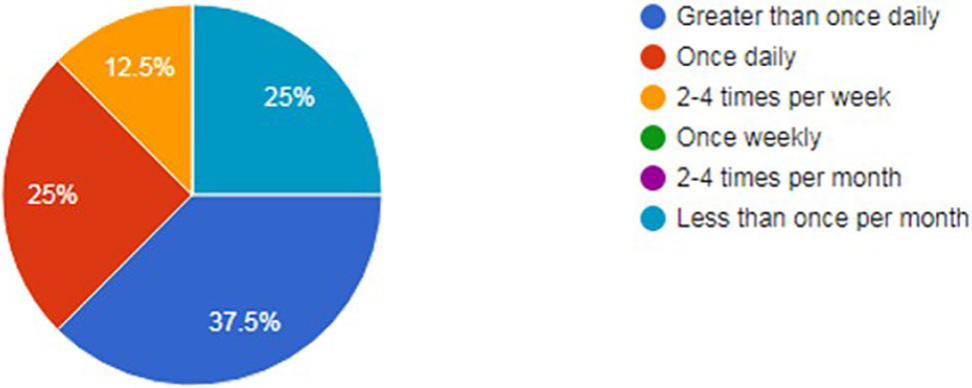
How often residents viewed their online profile.

**Figure 5.**
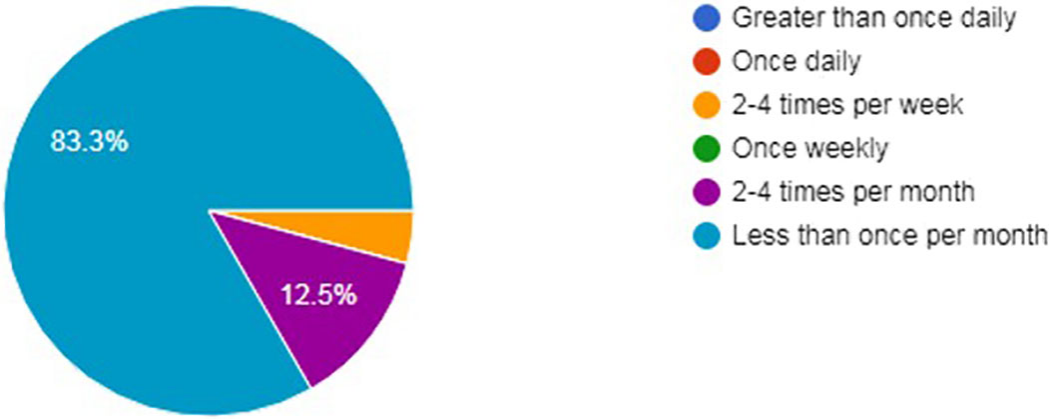
How often residents make changes to their online profile.

With regards to how often residents viewed their profile, approximately 52.5% viewed it at least once daily ( Figure 4). However, 83.3% of residents made changes to them less than once per month ( Figure 5). There was no statistical difference between age and how often residents viewed (p=0.827) or changed their profile (p=0.934).

**Figure 6.**
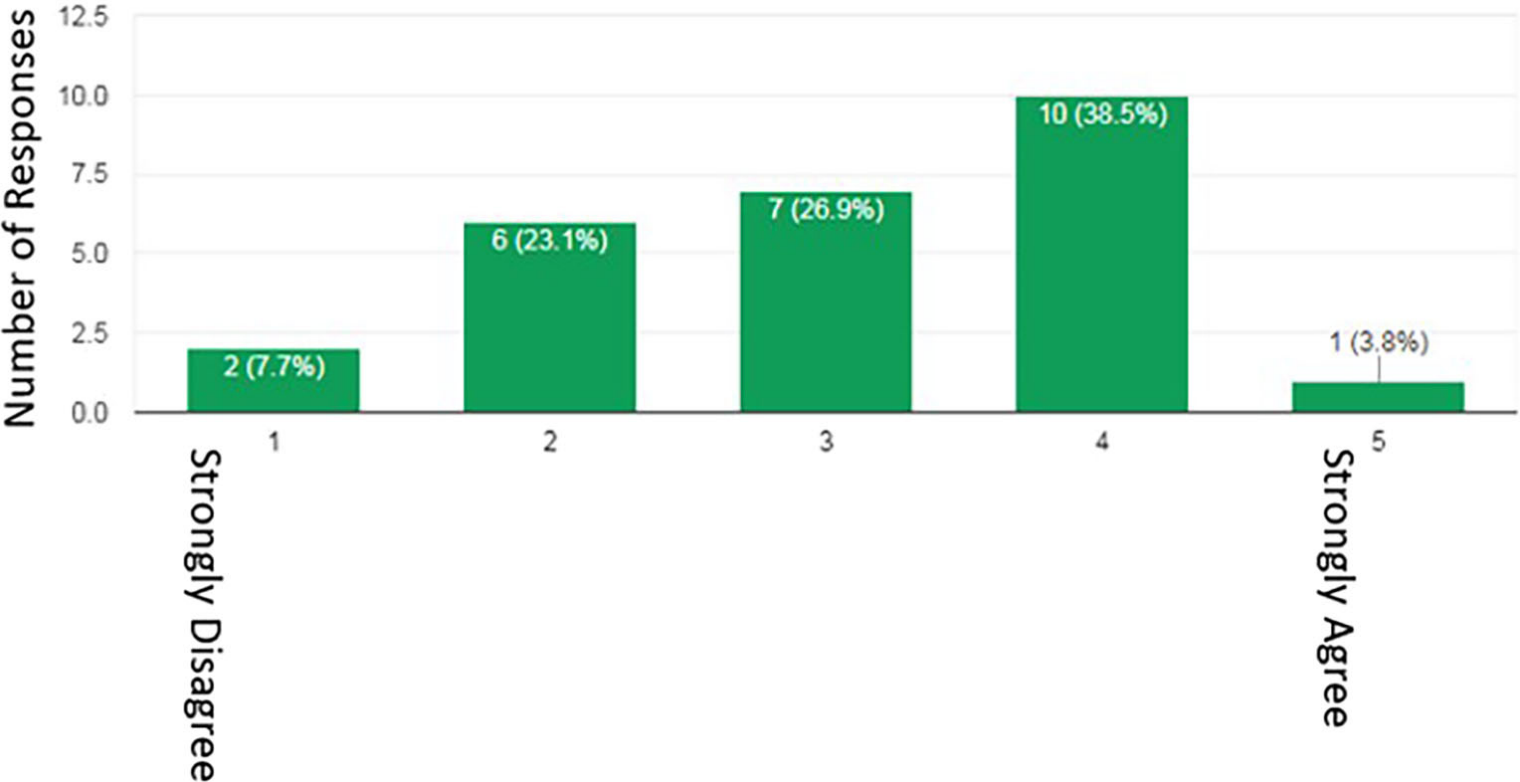
Do residents think Fellowship Selection Committees should use their online profile as part of the selection process?

**Figure 7.**
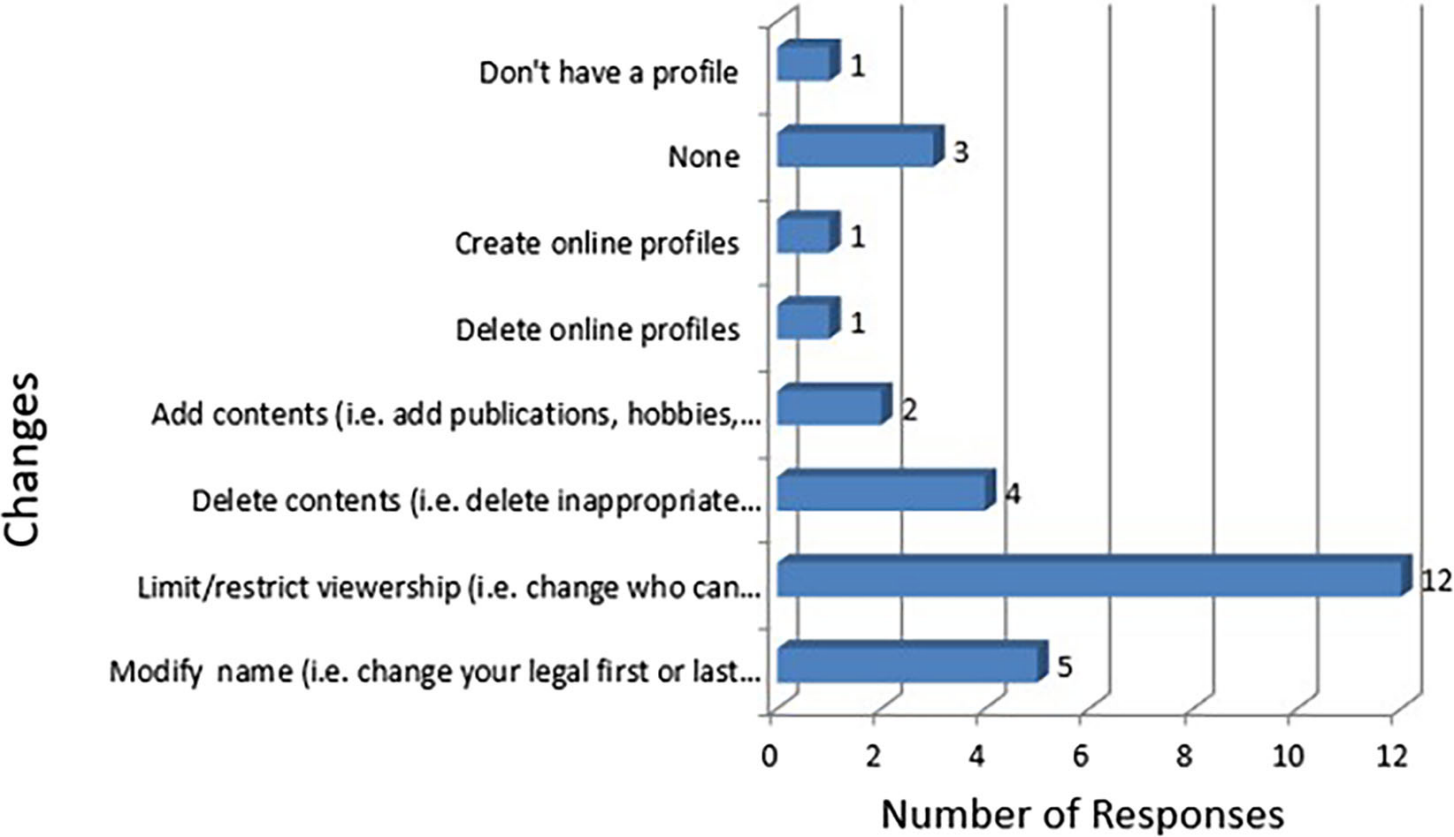
Changes that residents would make to their online profile for Fellowship applications.

When residents were asked if they think Fellowship Selection Committees assess their online profile as part of the selection process, 53.8% felt that they do, 42.3% were unsure and 3.8% felt that they did not. However, 69.2% of residents either agreed or were neutral about the committees doing so ( Figure 6). The age of the resident did not affect whether he/she agreed with their profile being used by Fellowship Selection Committees (p=0.91). However, 70% (n=14) would alter their profile for fellowship applications. 70.6% (n=12) would limit or restrict viewership of their profile so that they could change who would be able to view it. 29.4% (n=5) would change the name on their profile so that it was more difficult to identify their profile ( Figure 7). Of the respondents who would make changes (n=14), 13 (92.8%) would make changes at least 2 months prior to the fellowship application deadline. All those who would make changes would reverse or would not reverse the changes after securing a fellowship position. The majority of the remaining respondents who would not make changes (n=6), have stated that they have few/no online profile or have already restricted their online profile. When asked why residents would alter their profile, the majority said that it was due to the personal and sensitive information on their profile. Only 16.7% of residents would make changes to primarily reinforce their application.

## Discussion

The study’s primary goal was to determine the online profile of Canadian Diagnostic Radiology residents at our institution. Almost all Radiology residents at the University of Alberta had an online profile, which was consistent with radiology trainees in U.S.A and other specialties, including Family Medicine and Internal Medicine, where greater than 90% utilized social media ( Koontz, 2018). This online profile consisted of at least Facebook in the majority, which was consistent with a study evaluating Radiology residents in U.S.A. ( Capps, 2017). There was no statistical difference between age and having a profile, agreement with Fellowship Selection Committees using a resident’s profile for selection, how often residents viewed or changed their profile. This may be because all the residents who responded were between the ages of 25-33 years old, which would put them in “Generation X/Millennials,” and therefore were likely exposed to the same cultural influences. For example, being exposed to social media at a young age and being more receptive to it. This was suggested in a study by Go (2012), where 49% of program directors less than or equal to 50 years old (Generation X/Millennials) reported visiting applicants’ online profile, whereas only 5.4% of program directors greater than 60 years old did so.

With the awareness medical trainees have of the implications of their online profile and the increase use of it by hiring managers in other fields, medical trainees have noted that they believe selection committees visit their online profile. In the literature, Go (2012) found that less than 25% of medical students believed program directors view their online profile. In another study by Strausburg (2013), 70.5% of medical students believed that program directors assess their online profile and 60.1% of students were neutral or agreed with this. Moreover, 60.1% would alter their online profile before the residency application process with the majority making changes that would make it difficult for the selection committee to find their profile, including deleting (12.7%), altering privacy setting (28.2%), and altering the content (46.4%). In our study, the majority of residents perceived that Fellow Selection Committees assess their online profile, while a slightly lower number were unsure. The variance in the perception that selection committees use an applicant’s online profile exemplifies the infancy and lack of literature in this area. The ethical implications associated with this have also been debated, such as being able to separate personal and professional identities, and was suggested in our study by the fact that none of the residents used their online profile solely for professional use. The majority of trainees were neutral or agreed with their online profile being assessed. This may be because they have become accustomed from increased awareness and the increasing use of it in other jobs. Alternatively, it may be because the majority of residents plan to alter their online profile to make it difficult for selection committees to find, also seen in our study. Of those who didn’t make changes, they had already restricted who could view their profile or they didn’t have an online profile to begin with. This could be because of the potential repercussion of online profiles on their medical career from increased awareness or due to formal guidelines and statements from institutions and societies regarding social media. Interestingly 2 residents stated that they would make changes to reinforce their application and 1 would create online profiles, suggesting that applicants can also use their online profile to reinforce their application.

In the literature the intent of using an applicant’s online profile is to assess their clinical competence and professional accountability. For Fellowship Selection Committees, this may be of limited value given that the changes residents would make, would make it difficult for committees to identify their online profile and to obtain valuable added information. Furthermore, the fact that most residents will make changes 2 months prior to the application deadline would add additional challenges as the committees wouldn’t know which residents would apply to their program before changes are made. Therefore, whether this assessment tool would be an effective use of resources for Fellowship Selection Committees would have to be questioned.

There are several limitations with this study. The University of Alberta has a combined Diagnostic Radiology and Nuclear Medicine residency program, in addition to the stand-alone Diagnostic Radiology program. Residents who are in this combined program complete their Diagnostic Radiology training prior to transitioning to Nuclear Medicine. They do not apply for fellowships and would not be incentivised to make changes to their online profile. Furthermore, this was a single institution study. Internationally, there may also be differences in online profile usage, perception of Fellowship Selection Committees, etc.

A future study surveying multiple Canadian institutions from both languages may allow greater inferences. Since there are numerous radiology fellowship subspecialties available, sub-analyses may provide greater insights as to the residents’ behaviour when applying for more competitive subspecialties (residents who apply to non-competitive specialties may be less likely to alter their online profile when applying). Lastly, it would be prudent to survey whether Fellowship Selection Committees assess their applicants’ online profile.

## Conclusion

Nearly all Canadian residents at our institution had an online profile and over half viewed it at least once daily, but rarely made changes to it. The majority of residents perceived that their profile is assessed by Fellowship Selection Committees, but are not against it. However, in anticipation, residents would alter their online profile prior to the fellowship application deadline to make it difficult for their online profile to be identified. This was done mainly due to the sensitive and personal information on their profile.

## Take Home Messages

• Nearly all residents have an online profile. Facebook was the most popular.
• Over half viewed their online profile at least once daily, but seldom make changes to it.
• The majority of residents perceived that their profile is assessed by the Fellowship Selection Committees and would alter their online profile prior to the application deadline.

## Notes On Contributors

Anthony Vo, MD- a Diagnostic Radiology resident at the University of Alberta, Canada. He has an interest in medical education and using innovative approaches to enhance the learning experience of medical trainees.

Arlene Kanigan, MD- a Diagnostic Radiologist and Program Director of the University of Alberta Diagnostic Radiology Program.
