# Supplementary material for: Online Profile of Canadian Diagnostic Radiology Residents: Do Residents Alter Their Profile When Applying for Fellowships?
Source: MedEdPublish (2016). 2018 May 16;7:102. [Version 1] doi: 10.15694/mep.2018.0000102.1 (PMC10699382; doi:10.15694/mep.2018.0000102.1)
Supplement: Peer review history

# Reviewer report: Reviewer response for version 1

**DOI:** 10.21956/mep.19559.r29003

**Published:** 2019-05-08

**Authors:** Trevor Gibbs

**Affiliations:** 1 AMEE

This review has been migrated. The reviewer awarded 4 stars out of 5 An interesting but unusual paper that looked at an interesting subject. I feel that it does reflect the uncertainty , in the eyes of applicants, of how their personal data is interpreted. It was very interesting to see how many applicants were prepared to alter their profile. I do agree that this is a relatively new phenomenon and reflects the increasing use of social media and not just the reliance upon it but how well personal data is easily shared- not something that you would agree with either one or two decades previously. This is an important paper for those involved in selection procedures

Reviewer Expertise:

NA

No decision status is available

# Reviewer report: Reviewer response for version 1

**DOI:** 10.21956/mep.19559.r29002

**Published:** 2019-02-05

**Authors:** Ken Masters

**Affiliations:** 1 Sultan Qaboos University

This review has been migrated. The reviewer awarded 4 stars out of 5 A small but really useful study, nicely situated within the literature and medical context.Although it is known that companies view applicants’ online profiles, the extent to which medical schools do this is a little hazy (although this paper also gives some insight into that figure, and the impact of the viewing.)Those medical schools that do this, however, need to take note of a study like this, and be prepared for the fact that prospective residents may tailor their profiles particularly to meet perceived criteria.I am interested to know why the researchers decided on p Minor point: • “anonymous data was evaluated” should be “anonymous data were evaluated”Nice paper.

Reviewer Expertise:

NA

No decision status is available

# Reviewer report: Reviewer response for version 1

**DOI:** 10.21956/mep.19559.r29001

**Published:** 2018-06-01

**Authors:** David Bruce

**Affiliations:** 1 None

This review has been migrated. The reviewer awarded 4 stars out of 5 As a very limited user of social media, I read this paper with great interest. The blurring of professional and personal information that can result from use of social media and impact on career prospects is increasingly important. In this well designed survey we find almost all Alberta radiology residents have an online profile which is mostly for personal use. However, surprisingly to me, the residents mostly considered use of their profiles by fellowship selection committees as acceptable and accordingly made modifications as they felt needed. If such information, with a non standardised mix of personal and professional detail, is used by selection committees then the question of potential bias needs to be considered. Perhaps a companion paper looking at what information selection committees access and what guidance they are given would complete the picture. This paper should be read by all trainees and those taking part in the selection process.

Reviewer Expertise:

NA

No decision status is available
